# Twitter sentiment analysis: An Arabic text mining approach based on COVID-19

**DOI:** 10.3389/fpubh.2022.966779

**Published:** 2022-10-10

**Authors:** Saleh Albahli

**Affiliations:** Department of Information Technology, College of Computer, Qassim University, Buraydah, Saudi Arabia

**Keywords:** public health, sentiment analysis (SA), natural language processing, machine learning - ML, Synthetic Minority Over-sampling Technique (SMOTE)

## Abstract

The 21st century has seen a lot of innovations, among which included the advancement of social media platforms. These platforms brought about interactions between people and changed how news is transmitted, with people now able to voice their opinion as opposed to before where only the reporters were speaking. Social media has become the most influential source of speech freedom and emotions on their platforms. Anyone can express emotions using social media platforms like Facebook, Twitter, Instagram, and YouTube. The raw data is increasing daily for every culture and field of life, so there is a need to process this raw data to get meaningful information. If any nation or country wants to know their people's needs, there should be mined data showing the actual meaning of the people's emotions. The COVID-19 pandemic came with many problems going beyond the virus itself, as there was mass hysteria and the spread of wrong information on social media. This problem put the whole world into turmoil and research was done to find a way to mitigate the spread of incorrect news. In this research study, we have proposed a model of detecting genuine news related to the COVID-19 pandemic in Arabic Text using sentiment-based data from Twitter for Gulf countries. The proposed sentiment analysis model uses Machine Learning and SMOTE for imbalanced dataset handling. The result showed the people in Gulf countries had a negative sentiment during COVID-19 pandemic. This work was done so government authorities can easily learn directly from people all across the world about the spread of COVID-19 and take appropriate actions in efforts to control it.

## Introduction

The world went into a state of emergency in December of 2019 when the COVID-19 virus (coronavirus) started spreading, causing a lot of hospitalizations and, in many cases, death (1, 2). It is a virus that attacks the respiratory system of an infected person, causing shortness of breath and eventually inability to breathe (3). Governments are making an effort to stop the COVID-19 pandemic by applying lockdowns and long-term restrictions to limit the spread of infection. While on lockdown, people expressed their opinions, got information and news, and communicated mostly online through social media, especially Twitter. Twitter is a social media platform where people can compose a text message called a tweet, and the netizen can gather information from the tweet and retweet the message. Some people saw social media as an avenue to propagate the spread of false information, thereby fulfilling their agendas (4). Twitter has a comprehensive privacy policy and the content is genuine compared to other social media tools. The good thing about Twitter is it comes directly from people without any modification or agenda, so using it to discover, analyze, and track socio-political events is most effective (5). It is easy to understand user's opinions on all matters ranging from natural disasters, terrorists activities, or political inclination (6).

Sentiment analysis (opinion mining) involves breaking down text to find meaning; this proposed model uses Arabic text. It extracts useful information by extracting thoughts, opinions, and feelings from a sample of text data using Machine Learning or Deep Learning techniques (7). Twitter is the leading social media platform where most people communicate worldwide, including the Arab world. It skyrocketed in use during the pandemic because of the lockdown (8). Several studies about deep learning for sentiment analysis use LSTM (9). And also, there is a sentiment analysis of the Arabic language (8). Dealing with the data, Twitter is more of a broadcast medium for individual shout-outs (10). And for labeled data, Twitter also has a problem with an imbalanced dataset. The problem of imbalanced class distributions in opinion mining is vital for classification or sentiment analysis using Machine Learning and must be handled appropriately (11).

### Effects of COVID-19 on social media

It proves that most information being shared on social media is not true. The fact is that everyone is free to post what they want without any obligation to get the facts straight. And as the number of users increase, there is widespread misinformation, mostly from conspiracy theorists, which often leads to social unrest. Even though social media has numerous advantages, misinformation poses a significant threat to its credibility.

The spread of wrong information has proven to cause even more damage than the pandemic itself (8). Many researchers have taken this as a call to action as they began efforts to mitigate this problem of misinformation. Zhang et al. (9) conducted a study showing the reasons behind spreading false information, which ultimately increased truthfulness on social media by testing an intervention on Twitter. Kouzy et al. (10) studied the information on Twitter to determine their quality, to find why there is an increase in the belief of false information and claims, and learn how to correct them. Al-Azani et al. (11) studied 255 pieces of false information and categorized them according to their source, type, and claims. Then they reported simplified results intended to improve the effectiveness of countering the spread of misinformation. Al-Ayyoub et al. (12) confirmed the spread of political conspiracies in the United States using automated bots setup by conspiracy theorists during the COVID-19 pandemic. Elhag et al. (13) did a sentimental analysis of people's political views on the pandemic on Twitter to determine if they were positive or negative.

### Natural Language Processing

Natural Language Processing (NLP) is a useful approah for text dataset including linguistics. This approach is key to working on sentiment analysis. NLP techniques into sentiment analysis include tokenization of the sentences, lemmatization, and stemmer of the words. There approaches are suggested in the proposed method. Based on these approaches, it get the sentiments inside the text data could easily be obtained. Lexical analysis is the approach which is best fit to sentiment analysis as well. In this proposed model, only the above-mentioned approaches are used (9, 10).

### Research motivation

The proposed model explores sentiment analysis of COVID-19. Arabic tweets need sentiment analysis because there was no work previously done on this language before COVID-19, so this proposed model worked on this dataset. This proposed model analyzes the sentiment of Arabic texts tweeted in the Gulf countries during the COVID-19 pandemic using Machine Learning and SMOTE for imbalanced dataset handling. The study would also play a vital role in assisting the government in implementing guidelines in various regions based on how the region's people behave. Psychological assistance is also provided to citizens, which is crucial in a pandemic as many people are affected emotionally and mentally.

The rest of this paper has the following arrangement. Section “Related works” contains the literature review of existing techniques for analyzing people's sentiment during the COVID-19 pandemic and about SMOTE for imbalanced datasets. The proposed model is explained with detailed elaboration in section “Proposed method”. Then, the experiment is carried out and results obtained in section “Experiment and results.” The discussion and the limitations are covered in section “Discussion and limitation”. Finally, section “Conclusion and future studies” is the conclusion of the paper, and it states critical findings from the experiments and suggests works and improvements that could be made in the future to address these tweets and public opinions more effectively.

## Related works

There have been few research works available for sentiment analysis of Arabic language datasets with applied machine learning and deep learning approaches. Many research papers were published in different languages for sentiment analysis, but there was a need to address the same problem using the Arabic tweet dataset. This research study revealed many approaches like Natural Language Processing (NLP), Text Mining, and Machine Learning, reviewed in detail below.

Al-Ayyoub et al. (12) conducted several pieces of research using the Arabic language corpus. Opinion Corpus for Arabic (OCA) consisted of reviews from 500 Arabic movies. This research used Support Vector Machine (SVM) and Naïve Bayes (NB), which are Machine Learning algorithms, to predict classification of positive and negative reviews. Elhag et al. (13) used a systematic mapping study for Arabic Sentiment Analysis (ASA). This research showed the publication trend of the papers, and its dataset used 277 papers matched the search expression. Al-Smadi et al. (14) analyzed Arabic hotel reviews by applying Aspect-Based Sentiment Analysis (ABSA) using SVM, Decision Tree, Naïve Bayes, and K-NN (K Nearest Neighbors) algorithm in WEKA Classifier. For true positive prediction, the result is K-NN performs better than the SVM, around 85.3%. The imbalanced distribution of the prediction dataset class influenced this result greatly.

Gamal et al. (15) implemented Machine Learning algorithms using Naïve Bayes, Multinomial Naïve Bayes, Adaptive Boosting, Logistic Regression, Stochastic Gradient Decent (SGD), PA, RR, and SVM for sentiment analysis of social media platforms in the Arabic language. The research also employed N-Gram features to increase the accuracy. This research also used 151,500 tweets which are labeled correctly. The result showed the combination of PA and RR using unigram, bigram, or trigram has the best accuracy. Mohammad et al. (16) applied Machine Learning algorithms such as SVM, Naïve Bayes, and Multilayer Perceptron-Neural Network to an in-house created dataset. The result proves that SVM is better than Naïve Bayes and MLP-NN, obtaining 77.8% precision.

Elnagar et al. (17) used deep learning to perform text classification on Arabic text. This research used single-label SANAD and multi-label NADiA datasets. They used word2vec embedding models combined with convolutional-GRU and attention-GRU. The “Masrawy” dataset was made to have a maximum subset of 10 categories; the SANAD performed better with an accuracy of 96.4%, while the attention-GRU got an accuracy of 88.64%.

Sentiment analysis was also used in another language. Farisi et al. (18) implemented the Multinomial Naïve Bayes Classifier method for classifying positive and negative opinions using the Finitis Business Database of hotel reviews. This dataset contains 5,000 hotel reviews in English sentences. The dataset is manually labeled. Before implementing Multinomial Naïve Bayes, the data was done in preprocessing, feature extraction, and feature selection. As a result, Multinomial Naïve Bayes achieved an F1-Score of more than 91%. Suppala and Rao (19) also used Twitter data for sentiment analysis. They used Naïve Bayes Classifier to classify the Twitter data into positive and negative sentiments. The dataset, which contains 65,536 tweets, was collected. At the same time, Ruz et al. (20) proposed a method that can predict the sentiment during critical events. They used Bayesian Network classifiers for the task of sentiment analysis using the two datasets they have. These datasets are in Spanish and include the Chilean earthquake dataset of 2010 and the Catalan independence referendum of 2017. The result showed Bayesian networks classifiers were more effective than SVM and Random Forrest. And for the imbalanced dataset, they used SMOTE.

For imbalanced datasets, SMOTE was also used in several pieces of research. Ruz et al. (20) used SMOTE to train datasets to avoid class imbalance. This caused the dataset to notice more than 80% of the tweets were labeled negative. SMOTE adds synthetic samples with feature similarities with the aim of over-sampling the minority class between existing data. Al-Azani et al. (11) conducted sentiment analysis on short Arabic text and used Synthetic Minority Over-sampling technique (SMOTE) because the dataset was highly imbalanced. The result showed a 15% improvement in the average F1 score when the ensemble was combined with SMOTE for applied word embedding. Flores et al. (21) employed an evaluation of two classifiers, Naïve Bayes and SVM, with SMOTE technique on sentiment analysis. The dataset used Duterte Administration Tweets. The results clearly show that SMOTE positively affects the performances of Naïve Bayes Classifiers and Multinomial Naïve Bayes.

Bhatia et al. (22) discussed how social media services' use spread in the Arab countries. Due to COVID-19, the ways in which people used social media changed in Arab countries, specifically people using Twitter as their main source of news. This paper addressed the spread of COVID-19 in Arab countries and also gave the solution to use the different approaches for sentiment analysis. The goal of this study was to extract different regions in the Arab countries and then apply some preprocessing approaches to prepare the dataset for model training. Machine learning and deep learning approaches were used to get the sentiments on Arabic tweets' dataset. The model was evaluated and got 84% accuracy value using DNN algorithm. This model outperformed as compared to other state-of-the-art studies to get the sentiments of the people only using text tweets.

Al-Hashedi et al. (23) highlighted the sentiment analysis problem during COVID-19 for the Arab countries. People needed to be aware in the pandemic so the number of people in Arab countries using Twitter as a tool to get the most recent authentic information increased. Twitter became the most useful social media platform all over the world. This research study was focused on how to get the sentiments during the COVID-19 situation for the Arab countries and proposed their approach. Pre-processing was applied to extract the Word2Vect for word embedding, and a pre-trained continuous bag of words model used. Machine learning algorithm Naïve bayes and ensemble approaches were used for the classification of tweets. The whole model was evaluated with and without SMOTE analysis. But the approach using SMOTE a outperformed and got an 84.61% accuracy value.

After analysis, many approaches were applied for sentiment analysis; this research study finds out the following major points which are addressed in the implementation. The major target was to address the sentiment of the people in the COVID-19 situation. Data mining approaches were used to gather a multilingual dataset. Experiments were carried out on multiple to obtain a wide range of knowledge of the pandemic condition. Natural Language Processing (NLP) was used to authenticate the correct information on social media when applied to different approaches to get the lexical text data. Statistical analysis was necessary to get the data insights. Machine learning, deep learning, and ensemble learning approaches were also used in the sentiment analysis. This proposed model designed the model in the next section to implement the approach for sentiment analysis using an Arabic tweets dataset. Table 1 shows a summary of the sentiment analysis approaches developed in the literature.

**Table 1:** State-of-the-art survey for sentiment analysis approaches.

**Paper**	**Year**	**Approach**	**Dataset**
Al-Ayyoub et al. (12)	2019	SVM and Naïve Bayes	Arabic Twitter Dataset
Elhag et al. (13)	2019	Systematic Mapping Study	Arabic Sentiment Analysis (ASA)
Al-Smadi et al. (14)	2019	Aspect-Based Sentiment Analysis (ABSA) using SVM, Decision Tree, Naïve Bayes and K-NN (K Nearest Neighbors) algorithm in WEKA Classifier	Analyzed Arabic Hotel reviews
Gamal et al. (15)	2019	Machine Learning algorithms using Naïve Bayes, Multinomial Naïve Bayes, Adaptive Boosting, Logistic Regression, Stochastic Gradient Decent (SGD), PA, RR, and SVM	Arabic Twitter Dataset
Hamdan et al. (16)	2018	Machine Learning algorithms such as SVM, Naïve Bayes, and Multilayer Perceptron-Neural Network	In-house Created Dataset.
Elnagar et al. (17)	2020	Deep learning, single-label and multi-label SANAD	Arabic text, NADiA datasets
Farisi et al. (18)	2019	Multinomial Naïve Bayes Classifier	Finitis Business Database of hotel reviews
Suppala et al (19)	2019	Naïve Bayes Classifier	Twitter data
Ruz et al. (20)	2020	SMOTE to train datasets to avoid class imbalance	Arabic Tweets Dataset
Flores et al. (21)	2018	Naïve Bayes and SVM, with SMOTE technique	Duterte Administration Tweets Dataset
Surbhi et al. (22)	2022	Machine Learning and Deep Learning (DNN)	Arabic Tweets Dataset
Abdullah et al. (23)	2022	Naïve Bayes, Ensemble Approach and SMOTE	Arabic Tweets Dataset

## Proposed method

**Figure 1:**
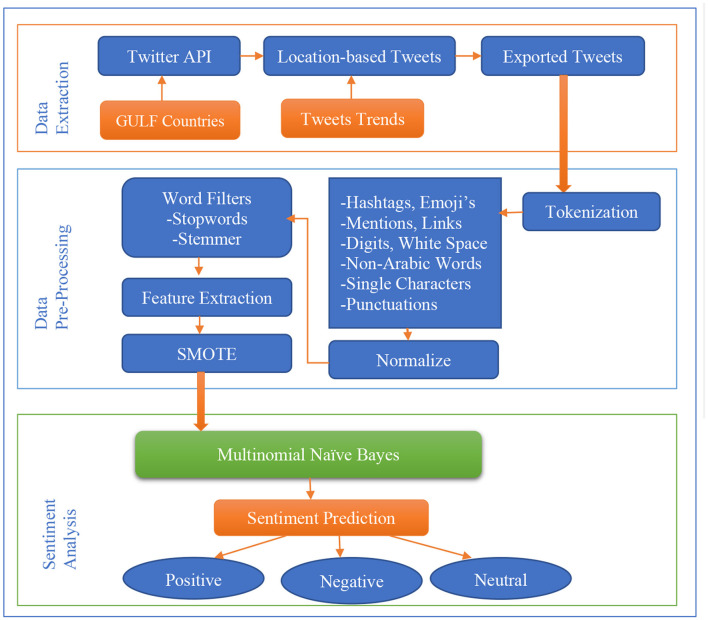
The proposed sentiment analysis pipeline.

This research aims to analyze the sentiment of Arabic text related to COVID-19 in Gulf Countries using Machine Learning and SMOTE for imbalanced dataset handling. The dataset comprised Arabic tweets posted at an interval during the COVID-19 pandemic. The data used for this work only focused on the gulf countries, i.e., Oman, Qatar, Bahrain, Saudi Arabia, and United Arab Emirates (UAE). Figure 1 shows our sentiment analysis architecture. It also shows the proposed model pipeline for the implementation. There are three major portions of the proposed method which are clearly shown: data extraction, data pre-processing, and sentiment analysis.

### Data extraction

The dataset was collected from Twitter from the beginning of March to the end of April 2020. In addition, the place filter was specified, so the only tweets extracted were from the aforementioned Gulf countries. The raw Arabic Twitter data was extracted using the Tweepy library and Twitter API. Afterward, the dataset was collected in CSV format. This dataset was separated into different files for each of the Gulf regions. The CSV files also included the information of individuals posting tweets, dates, and countries. The total dataset is 60,000 tweets collected using the keywords corona, coronavirus, COVID, covid19, and sarscov2.

### Data pre-processing

The data acquired from the Twitter API is not in real-time but rather a collection of tweets over a course of time. The dataset could possibly contain duplicate tweets. To ensure that we remove all the duplicate tweets from our data, data cleaning was carried out, and resulted in 33,496 tweets of all the countries combined.

After cleaning data in the first stage, there is a need to apply another cleaning technique to remove the non-required content of the dataset before tokenizing the Arabic text data. Tokenization is the process of breaking down text into tokens, the smallest units of the text. There are various tokenizers for text data, and each has its meaning of the smallest unit. In most cases, words, sentences, numbers, and even punctuation are considered the smallest units or tokens. After we apply tokenization, we remove hyperlinks, mentions, emojis, hashtags, foreign words, digits, punctuation marks, and single characters. We used Natural Language Toolkit (NLTK) library (24) for tokenization. NLTK performs the tokenization and the Arabic tweets are converted into the tokens. NLTK works the same in Arabic as it works for other language's tokenization.

The spam tweets treated in this phase were those with unnecessary content which is not in the same pattern. These were removed and the cleaned tweets passed to the model for feature extraction and then for classification. The different tweet patterns were filtered out and only the tweets which have a different nature were passed because Arabic language has patterns that helps to identify tweets.

The dataset contains tweets posted by users spread in different regions. Thus, the Arabic text depends mainly on the region's aesthetics and style. Consequently, the collected data is not homogenous. Therefore, we apply data transformations to make our data homogenous. This allows to standardize and normalize the text at later stages of our work. The Arabic Language is unique, with most of its letters taking contextual letterforms such as (أ إ آ). When we come across such, we normalize it to (ا). Also, the diacritics (َ ً ُ ٌ ِ ّ) that help in the pronunciation of letters properly are not needed and are therefore removed as well.

Afterward, we removed the stop words, which are common words with no meaning but are needed for sentence structure (for instance, هذا, الذين, ليت, هو). After removing the stop words, we stemmed the cleaned data. Stemming involves the reduction of words in their root form to word stems, e.g., (الكتاب, يكتبون, تكتبين) are converted to (كتب) after stemming. For stemming the Arabic text, we used ISRI Arabic Stemmer (25) that is available in the Natural Language Toolkit (NLTK) library.

Afterward, we split the dataset for training and testing into 70 and 30%, respectively. Then we used Bag-of-Words, a technique for feature extraction. Bag-of-Words is a method for sentiment analysis where sentences are broken down into words based on relationships. Every word determines the Arabic sentiment analysis and those values are united.

Normalization was the process which obtained the tweets after tokenization and pre-processing the special characters. The obtained dataset were imbalanced as compared to the three classes. This normalization process works to balance the whole dataset so that the actual results can be obtained after execution of the whole model.

And lastly, a common problem is an imbalanced dataset, which we handled using Synthetic Minority Over-sampling Technique (SMOTE) (26). SMOTE is used to increase the number of cases in the dataset in a balanced way, meaning smaller classes are integrated with synthetic data to match them based on similarities. SMOTE will handle the Arabic Twitter labeled. In the dataset, the Arabic text with neutral, negative and positive has a different balance for each country. With SMOTE, the labeled data will be rebalanced. So, we will have a balanced dataset for training using a Machine Learning algorithm. If we were not balancing the data, the result would be similar to the most common sentiment in the dataset.

### Sentiment analysis

For sentiment analysis, Multinomial Naïve Bayes, a Machine Learning algorithm, was employed for classification. Multinomial Naïve Bayes was used to determine term frequency, which is the count of a term's occurrence in a text or document. Term frequency is also used to decide whether a term can be used in the analysis. Some terms, such as conjunctures, may be present in the text but do not affect the sentiment (18). Singh et al. (27) proved that Multinomial Naïve Bayes outperforms Bernoulli Naïve Bayes for sentiment analysis. Another research proposed by Ali et al. (28) studied the hierarchical structure of sentiment analysis in terms of finding correlation between tags to study multitag learning algorithms.

## Experiment and results

In this research, the proposed model worked on sentiment analysis for Arabic text in Gulf Countries using Machine Learning and SMOTE using Twitter data. To do this, Python3 and many libraries were used to process the complete execution. Python3 was employed for developing all of the codes for this sentiment analysis research. Then, we used the following Python libraries.

1) NLTK Library
2) Tweepy, a Twitter REST API for accessing tweets
3) Pandas Library
4) Imbalanced-learn (for handling imbalanced data using SMOTE)
5) Scikit-learn (for implementing Machine Learning algorithm).

**Figure 2:**
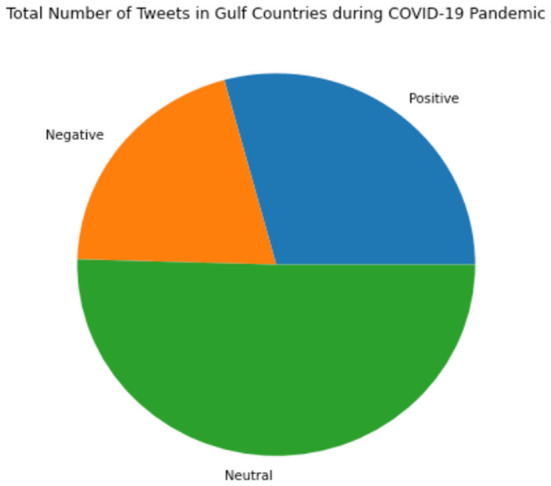
Total Number of Tweets in Gulf Countries during the COVID-19 Pandemic.

Figure 2 shows all the sentiments for the tweets that have been used. These tweets started at the beginning of March and continued to the end of April 2020 in the six countries mentioned above; this pie chart was drawn after all unnecessary words and symbols were removed to get precisely what the tweets portray. It can be seen that tweets with neutral intent have the highest labeled portion of the collected dataset, taking up more than half of the total tweets. In contrast, the positive and negative sentiments take up the rest of the portion, with positive being slightly larger than the negative.

In Table 2, we see the comparison of the positive, negative, and neutral sentiments in the dataset. It shows that Saudi Arabia almost has a neutral sentiment with around 3,737 Arabic tweets. The proposed model also followed with United Emirates Arab, Oman, Kuwait, Bahrain, and Qatar. Figure 2 shows more neutral values because during the pandemic, the dataset obtained from Twitter is mainly of neutral class tweets.

**Table 2:** Comparing the three sentiments of tweets from the Gulf countries during the COVID-19 pandemic.

**Country**	**Positive intent**	**Negative intent**	**Neutral intent**	**Total**
Saudi Arabia	2,597	1,552	3,737	7,886
U.A.E	1,250	1,897	3,560	6,707
Oman	1,563	961	2,053	4,577
Kuwait	1,385	771	2,187	4,343
Bahrain	1,723	984	3,040	5,747
Qatar	1,263	651	2,316	4,230

### Comparing three sentiments with gulf countries' dataset

After successfully executing the proposed model, the results were calculated using Arabic Tweets for sentiment analysis. The GULF datasets, Saudi Arabi, U.A.E, Oman, Kuwait, Bahrain, and Qatar, were used. Only text tweets of the following countries' datasets were collected and used to compute the results. Three sentiments were compared in these countries' datasets with positive intent, negative intent, and neutral intent. Table 2 shows the obtained results against these countries' tweets dataset, in which the total number of tweets was also shown.

**Figure 3:**
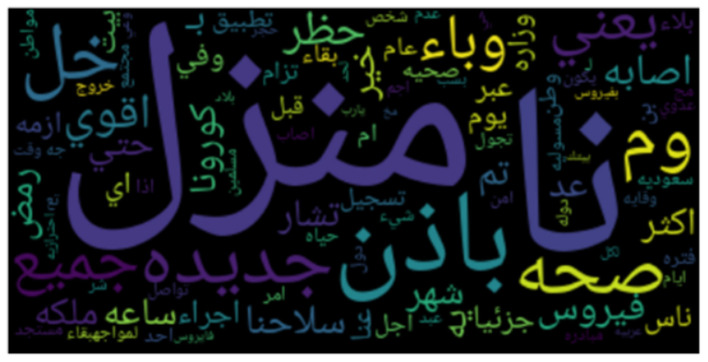
Word cloud of tweets from Saudi Arabia.

**Figure 4:**
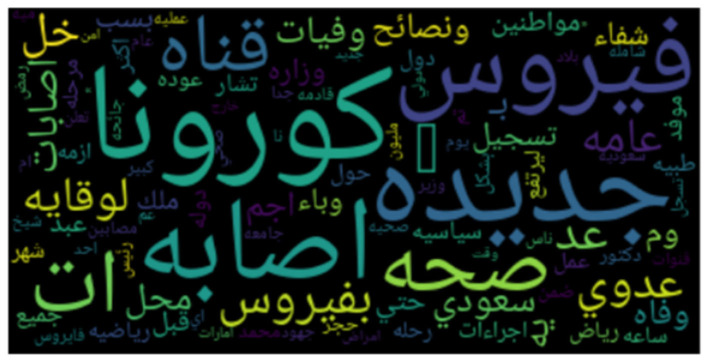
Word cloud of tweets from United Arab Emirates.

**Figure 5:**
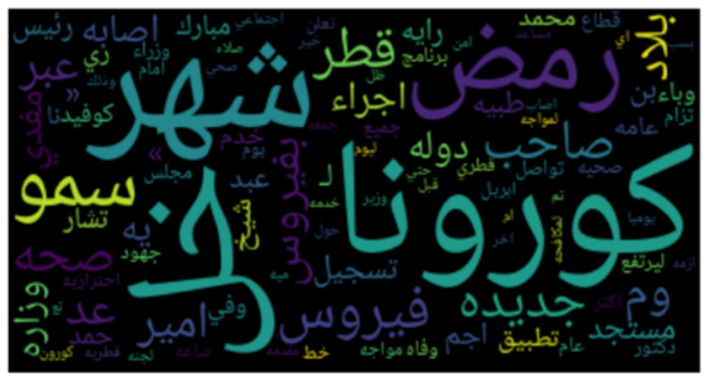
Word cloud of tweets from Qatar.

**Figure 6:**
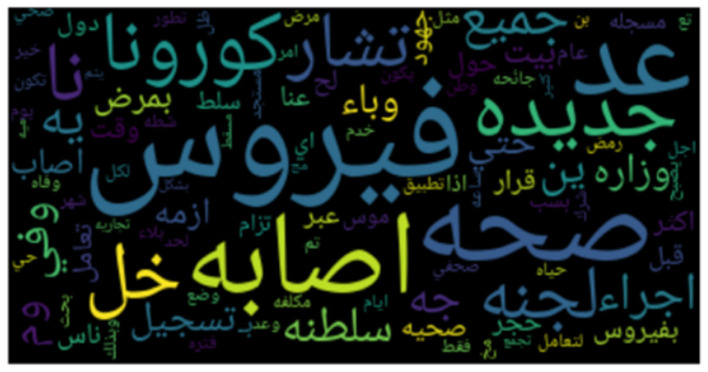
Word cloud of tweets from Oman.

**Figure 7:**
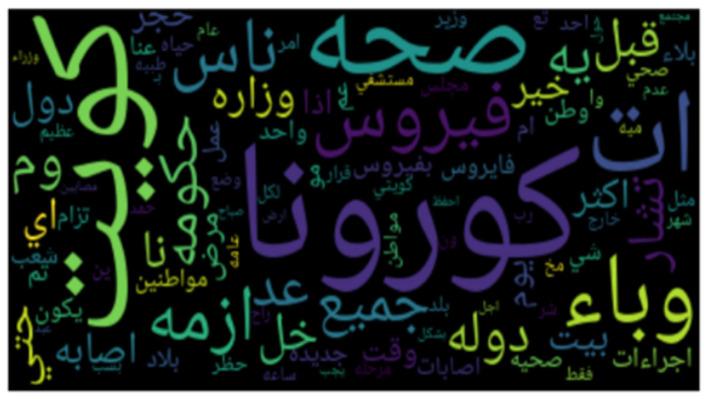
Word cloud of tweets from Kuwait.

**Figure 8:**
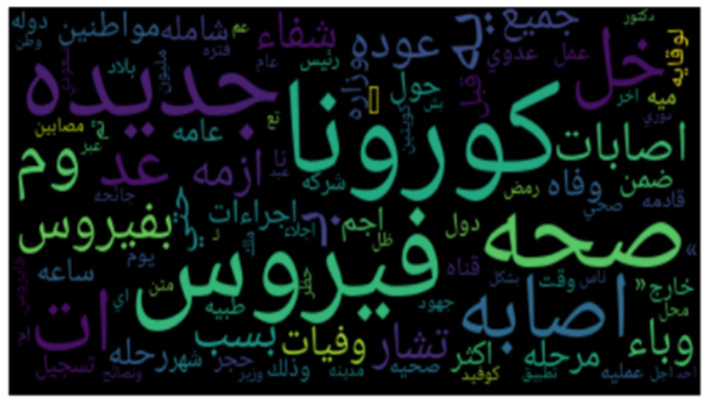
The word cloud of Bahrain.

**Figures 3**–**8** show the word clouds for all the regions. A word cloud or tag cloud is a form of data visualization for text data, and it depicts the most commonly used words with their intensity. The larger the word, the more frequently that word is used, while smaller words are words that have lesser frequencies. The figures show the most dominant word used in the tweets was “کورونا” which translates to “corona.” Another very frequently used word is “فیروس ,” meaning “virus.” We also see repeated use of “ وبا ء ” meaning pandemic or epidemic.

In Figure 3, we see the most commonly used words in Saudi Arabia are صحہ, اذن, وباء, منزل, حظر, which means “curfew,” “stay home,” “pandemic,” “permission,” and “Health ministry,” respectively. In Figure 4, we see the word cloud of UAE and it can be clearly seen that the most frequently tweeted words that translate to “Corona,” “virus,” “Saudi Health,” and “new cases,” respectively are اصابہ جدیدہ, الصحہ السعودية, فیروس, کورونا. In Figure 5 we can see the word cloud of Qatar and the most frequently used words which translate to “month,” “Ramadan (month of fasting),” “Corona/COVID,” “Application,” and “Prince or Sheik,” respectively are شھر, سمو, تطبيق, کورونا رمضان.

In Figure 6 we can see that the most frequently tweeted words in Oman which translate to “COVID,” “Crisis,” “new cases,” “Sultanate of Oman,” and “virus,” respectively are فیروس, سلطنة اصابہ جدیدہ, أزمه, کورونا,.. The most frequently used words people tweet from Kuwait is seen in Figure 7 and they are ازمة, کورونا, وباء, کویت, صحہ which means “Ministry,” “Kuwait,” “Pandemic,” “Corona/COVID,” and “crisis/chaos,” respectively. The most common words tweeted from Bahrain is clearly seen in Figure 8, the words are الحياة بعد كورونا, کورونا, فيروس, تفشي, وفيات, اصابہ which mean “new cases,” “Outbreak,” “deaths,” “virus,” “COVID,” and “Life after Covid,” respectively.

The initial stage was tokenization of the dataset, normalization and feature extraction, then applying SMOTE to handle the imbalanced data. Table 2 shows that most Arabic tweets have a neutral sentiment. We could not implement sentiment analysis if the dataset is not balanced. This is because it will improve the result. So, we proposed SMOTE method for handling the dataset. The total dataset after implementing SMOTE is shown in Table 3.

**Table 3:** The dataset after SMOTE was applied.

**Country**	**Positive**	**Negative**	**Neutral**
Saudi Arabia	2,623	2,623	2,623
U.A.E	2,471	2,471	2,471
Oman	1,443	1,443	1,443
Kuwait	1,540	1,540	1,540
Bahrain	2,129	2,129	2,129
Qatar	1,629	1,629	1,629

**Figure 9:**
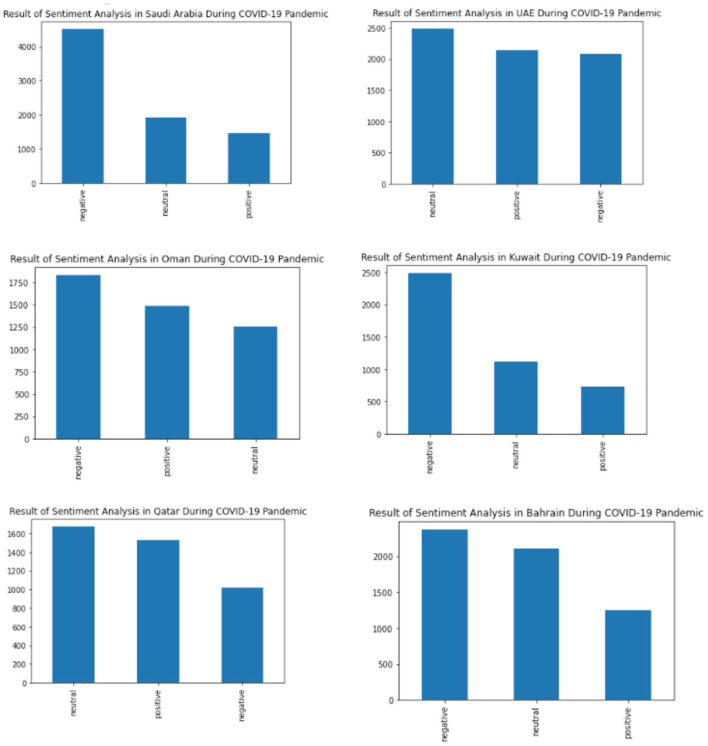
The results of Sentiment Analysis in Each Gulf Country.

The result of the sentiment analysis in Arabic tweets toward the global pandemic of COVID-19 opinion in Gulf Countries using Machine Learning and SMOTE is shown in Figure 9 and Table 4. In Figure 3, the highest result is Negative sentiment. From the Gulf Countries, the Negative sentiment consists of 14,311 tweets in Arabic, then the Neutral sentiment consists of 10,574 tweets, and the Positive consists of 8,605 tweets.

**Table 4:** The results of Sentiment Analysis in Gulf Countries during the COVID-19 pandemic.

**Country**	**Positive**	**Negative**	**Neutral**	**Total**
Saudi Arabia	1,452	4,513	1,921	7,886
U.A.E	2,140	2,076	2,491	6,707
Oman	1,490	1,832	1,255	4,577
Kuwait	733	2,491	1,119	4,343
Bahrain	1,257	2,378	2,112	5,747
Qatar	1,533	1,021	1,676	4,230
Total	8,605	14,311	10,574	

As shown in Figure 9, Saudi Arabia has a negative sentiment for the COVID-19 pandemic. It is also seen in Oman, Kuwait, and Bahrain. In United Emirates Arab and Qatar, the people tend to have a neutral sentiment. The details of the result are shown in Table 4. In Saudi Arabia, it is mostly a negative sentiment followed by neutral and positive sentiment. The negative sentiment of people in Saudi Arabia was around 4,513 tweets in Arabic Text followed by 1,921 in neutral and 1,452 in positive neutral. In United Emirates Arab, the peoples tend toward a neutral sentiment with around 2,491 tweets. This was followed by positive sentiment and negative sentiment. In Oman, most people have a negative neutral. The total negative sentiment in Oman consists of 1,832 tweets. Bahrain also has negative sentiments followed by neutral and positive. And in Qatar, the neutral sentiment was around 1,676, followed by positive around 1,533 and negative sentiment around 1,021.

The results obtained also show that most people in the Gulf region had negative sentiment during the pandemic.

### Comparison with state-of-the-art approaches

The proposed model was compared with state-of-the-art approaches to prove the quality. The proposed model used an Arabic tweets dataset for sentiment analysis (all details can be seen in Table 5). Multinomial Naïve Bayes algorithm was used for the classification between positive, negative, and neutral classes. According to the literature review performed in the related works, the other approaches used some different approaches but there was a need to address the Arabic tweets dataset specifically. An English language dataset was used for sentiment analysis on the COVID-19 but there was a lack of Arabic tweets dataset. The other reason for this study was to provide a solution to the COVID-19 situation, but there were only a few approaches used so there was still space for a solution in this area.

**Table 5:** Comparative analysis of the proposed model with state-of-the-art approaches.

**Reference**	**Dataset**	**Approach**	**Evaluation**
Manal et al. (29)	Arabic Tweets	Naïve Bayes (NB), Multinomial Naïve Bayes (MNB), K Nearest Neighbor (KNN), Logistic Regression (LR), and Support Vector Machine (SVM)	Accuracy 90%
Proposed Model	Arabic Tweets	Multinomial Naïve Bays	Accuracy 91%

### Major research contribution

The proposed model has major contributions to sentiment analysis method using machine learning and handling the problem of Twitter imbalanced datasets using SMOTE. The proposed model conducted the sentiment analysis using Arabic language, which has not been commonly done in Artificial Intelligence (AI) research. The proposed model can improve the past research using Arabic language on sentiment analysis for Twitter datasets. The result of this sentiment analysis showed how the pandemic affects people in Gulf countries. It can track the rise of panic and fear in people cause by COVID-19 by tracking the sentiment of their tweets. It can be used to examine behaviors and the new public lifestyle.

This research study helped people all over the Arab countries to know about the sentiment analysis for COVID-19. This research study also can be helpful for other countries and the same model can be applied to change the Arabic language dataset. The proposed model will contribute to studies analyzing the pandemic.

## Discussion and limitation

**Figure 10:**
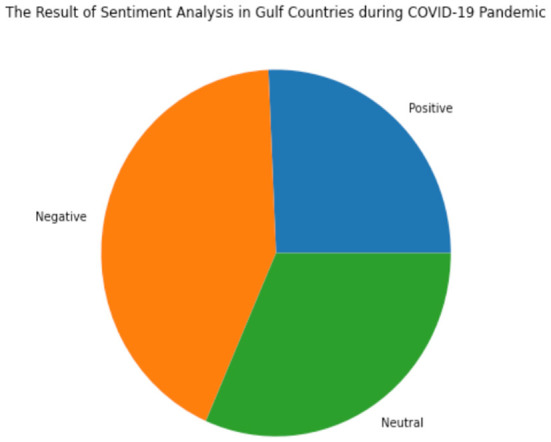
The result of Sentiment Analysis in Gulf Countries during the COVID-19 pandemic.

An analysis of a Twitter dataset was carried out to determine the sentiment of the tweets of people in Gulf countries. Each country's data was analyzed separately and results were compiled separately and collectively. Collectively speaking, it has been noticed that more than half of the population has a negative review of the COVID-19 situation. Because of this, the government should take measures to deal with those people as situations like this could lead to more significant problems such as a mental breakdown. The government should provide hotlines and online consultations as these might help handle the problems effectively. Another way of dealing with this issue is to control the misinformation on social media platforms; people might get scared of some misleading news. The government should also hold interventions to mitigate the problem of the spread of false information. It would also be helpful to know how the COVID-19 pandemic affected people in the Gulf countries directly from them. After carefully studying the collected data and looking at the time frame and how the pandemic affected the Gulf countries, we came to these conclusions:

The first was people present negative reactions at the beginning of the virus (March 2020), which should not come as a surprise because a pandemic causes a state of unrest ranging from panic attacks to stress and depression. This reflects the measures taken to effectively prevent and reduce the spread of COVID-19 in Gulf countries, such as closing state and national borders, enforcing social distance, restriction of movement by enforcing curfews, and imposing a virtual work model to control the spread of the virus. Next, a neutral reaction comes after the negative feeling is ascertained because of the increase in the number of people that contact the virus daily and the rising death rate. The people tend to be neutral towards the pandemic as shown in Figure 10. Lastly, a positive sentiment appears when the virus is more controlled. There is contributed to by any test controlling their spread.

The limitation of this research study was it only used the Arabic Tweets dataset and addressed the sentiment analysis. The sentiment analysis classification of the three classes was performed using only the GULF countries' dataset. The dataset was collected only from 1st March to 28th April 2020, almost 2 months. And the proposed model grabbed the data only from the six countries of the Gulf region: Saudi Arabia, United Arab Emirates, Oman, Kuwait, Bahrain, and Qatar. Noticeably, the datasets are manually labeled in positive, negative, and neutral. Emojis are not in the scope of this work. Hence, they were removed from texts that contained them. The research only focused on the result of the sentiment analysis and how to handle an imbalanced dataset.

## Conclusion and future studies

COVID-19 is a deadly disease that surprised the world and started spreading like wildfire. In this research, we performed sentiment analysis of Arabic text toward COVID-19 opinions using tweets in Gulf Countries using Machine Learning and SMOTE for imbalanced dataset handling. The collected data span from the 1st of March 2020 to the 28th of April 2020 and it was made up of tweets in Arabic. The coronavirus affected the world in ways we have never seen before. The purpose of this research is to highlight the reaction of the people of the Gulf countries. This is done by performing sentiment analysis on the Twitter posts from the people of these regions. We used Machine Learning to carry out a sentiment analysis on Arabic text and SMOTE for handling imbalanced data. The result is almost every country has a negative sentiment during the COVID-19 pandemic. Saudi Arabia, Kuwait, Oman, and Bahrain have a negative sentiment during this pandemic. But in United Arab Emirates and Qatar, most people have a neutral sentiment. The proposed model after applying the classification model Multinomial Naïve Bayes classified the sentiments into positive, negative, and neutral tweets. The proposed model mined the opinions of people in the Gulf countries using Twitter. The Arabic tweets dataset was used to enhance the sentiment analysis in the Arab countries.

For future work, big data is needed for sentiment analysis. More balanced data will get a better result. And also, there is a need for word embedding and pre-processing.

## Data availability statement

The original contributions presented in the study are included in the article/supplementary files, further inquiries can be directed to the corresponding author.

## Author contributions

The author confirms being the sole contributor of this work and has approved it for publication.

## Conflict of interest

The authors declare that the research was conducted in the absence of any commercial or financial relationships that could be construed as a potential conflict of interest.

## Publisher's note

All claims expressed in this article are solely those of the authors and do not necessarily represent those of their affiliated organizations, or those of the publisher, the editors and the reviewers. Any product that may be evaluated in this article, or claim that may be made by its manufacturer, is not guaranteed or endorsed by the publisher.
